# Correlative X-ray micro-nanotomography with scanning electron microscopy at the Advanced Light Source

**DOI:** 10.1107/S1600577524009305

**Published:** 2024-10-29

**Authors:** Arun J. Bhattacharjee, Harrison P. Lisabeth, Dilworth Parkinson, Alastair MacDowell

**Affiliations:** a Energy Geosciences Division Lawrence Berkeley National Laboratory 1 Cyclotron Rd Berkeley CA94720 USA; b Advanced Light Source Lawrence Berkeley National Laboratory 1 Cyclotron Rd Berkeley CA94720 USA; University of Malaga, Spain

**Keywords:** X-ray tomography, scanning electron microscopy, basalt, serpentinite, energy-dispersive X-ray spectroscopy

## Abstract

Geological samples are inherently multi-scale. Understanding their bulk physical and chemical properties requires characterization down to the nano-scale. A powerful technique to study the three-dimensional microstructure is X-ray tomography, but it lacks information about the chemistry of samples. To develop a methodology for measuring the multi-scale 3D microstructure of geological samples, correlative X-ray micro- and nanotomography were performed on two rocks followed by scanning electron microscopy with energy-dispersive spectroscopy (SEM-EDS) analysis. The study was performed in five steps: (i) micro X-ray tomography was performed on rock sample cores, (ii) samples for nanotomography were prepared using laser milling, (iii) nanotomography was performed on the milled sub-samples, (iv) samples were mounted and polished for SEM analysis and (v) SEM imaging and compositional mapping was performed on micro and nanotomography samples for complimentary information. Correlative study performed on samples of serpentine and basalt revealed multiscale 3D structures involving both solid mineral phases and pore networks. Significant differences in the volume fraction of pores and mineral phases were also observed dependent on the imaging spatial resolution employed. This highlights the necessity for the application of such a multiscale approach for the characterization of complex aggregates such as rocks. Information acquired from the chemical mapping of different phases was also helpful in segmentation of phases that did not exhibit significant contrast in X-ray imaging. Adoption of the protocol used in this study can be broadly applied to 3D imaging studies being performed at the Advanced Light Source and other user facilities.

## Abstract 2

A protocol for performing correlative micro-nanotomography with scanning electron microscopy analysis at the Advanced Light Source is described. The protocol was used for the analysis of basalt and serpentine mineral samples.

## 1. Introduction

Study of the microstructure of geological samples involves the identification of different mineral phases and pores contained within the samples. Size and morphology of these mineral phases provide insight into the history of the sample as physio-chemical processes form and transform these minerals over geological time. Pore networks present in such samples can be signatures of the pressure and temperature environment in which these minerals evolved and provide insight into the hydraulic and petrophysical properties of the material. These properties are essential for understanding the physical behavior and evolution of the subsurface. As such, 3D characterization tools have long been employed to analyze the mineralogy, porosity and fabric of geological samples.

One of the most commonly used techniques for 3D characterization of geological samples is X-ray computed tomography (CT) (Cnudde & Boone, 2013 ▸; Mathews *et al.*, 2017 ▸; Taina *et al.*, 2008 ▸). Depending on the X-ray absorption and attenuation by different phases present in a sample, it is possible to acquire X-ray images with appreciable contrast between different minerals and pores. X-ray computed tomography, being an inherently 3D technique, is capable of revealing the 3D morphology of different phases and their distribution within the sample that cannot be ascertained by traditional microscopy and scanning electron microscopy (SEM). It is also commonly used in determining the volume fractions and spatial distributions of different solid phases as well as porosity. With the aid of available X-ray tomography analysis software and algorithms, it is also possible to distinguish between isolated pores, pores that are connected over long distances and pores accessible from the sample surface.

Although laboratoy-based X-ray tomography systems are increasingly common, synchrotron X-ray tomography at multiple synchrotron facilities around the world is often used to take advantage of the high X-ray flux generated by these X-ray sources (Wang & Miller, 2020 ▸; Stock, 2008 ▸). The sub-micrometre resolution of synchrotron-based X-ray CT helps in identifying features that are as small as hundreds of nanometres. Another advantage of synchrotron X-ray tomography is the high coherence of the X-ray beam. Using a highly coherent beam and increasing the distance between the sample and detector it is possible to perform phase-contrast tomography for low-density materials or for samples that contain multiple phases where the absorption contrast is insufficient for segmentation (Mayo *et al.*, 2012 ▸). The high flux of X-rays generated by synchrotron sources decreases the scan time required per sample and thereby accomplishes high throughput of data, making statistical measurements possible. Recent advancements in data acquisition systems have also made it possible to perform reconstruction of the data as they are being acquired (Nikitin *et al.*, 2022 ▸). Shorter scan times of synchrotron tomography also opens the avenue of *in situ* experimentation, where the change occurring in the sample during the scan needs to be undetectable in order to obtain a good reconstructed image. In the last several decades, various apparatuses have been developed that are capable of changing the sample environment in order to perform *in situ* experiments. Induction furnaces along with laser and high-intensity lamp systems have been used for analyzing homogenization, melting and solidification phenomena in samples across multiple disciplines (Robinson *et al.*, 2014 ▸; Cai *et al.*, 2016 ▸; Bellet *et al.*, 2003 ▸). Compression and tension systems have also been developed to analyze failure mechanics (Buffiere *et al.*, 2010 ▸). Additionally, systems with fluid environments are also commonly being used to understand oxidation and corrosion behavior in samples (Ghahari *et al.*, 2011 ▸).

Synchrotron-based microtomography can be employed to perform 3D analysis of samples from several millimetres to centimetres in size, which is suitable for obtaining a good statistical understanding of the sample. However, the limitation of microtomography is its resolution, which is not comparable with other commonly used high-resolution 2D imaging techniques such as electron microscopy. A standard scanning electron microscope with nanometre-scale resolution can resolve features that are not detectable in microtomography reconstructed images. This problem can be solved by using synchrotron nanotomography, which employs a Fresnel zone plate as magnifying lens for the attenuated X-ray beam. The resolution of synchrotron nanotomography can range from 10 to 150 nm depending on the setup of the beamline (Wakabayashi *et al.*, 2022 ▸; Takeuchi *et al.*, 2021 ▸). Generally, the field of view (FOV) for nanotomography at a synchrotron source is also considerably smaller (20–100 µm), depending on the configuration of the zone plate used.

Features difficult to identify using nanotomography can be analyzed using electron microscopy. Correlative micro- and nanotomography with SEM becomes a powerful characterization protocol, which can reveal the finer details of the sample microstructure at various hierarchal levels (Wu *et al.*, 2017 ▸; Goral *et al.*, 2020 ▸; Okuma *et al.*, 2023 ▸; Huang *et al.*, 2023 ▸). Furthermore, energy-dispersive X-ray spectroscopy (EDS) in SEM can provide the elemental composition of the sample. Elemental distribution in a sample can also be acquired by synchrotron X-ray fluorescence (XRF) spectroscopy. For 2D XRF elemental mapping it is preferrable to have a thin slice of the sample, as the excited photon in XRF can originate from different depths in the sample (Borsato *et al.*, 2021 ▸; Chen *et al.*, 1993 ▸). A potential solution to this is to use XRF tomography which provides 3D elemental mapping of the sample (Lin *et al.*, 2024 ▸). However, a full 3D elemental map with high resolution can result in a significantly long scan time. If the compositional information of the sample needs to be acquired in non-destructive manner, XRF is a highly suitable technique. However, if the sectioning of the sample is acceptable, performing the compositional analysis using EDS provides better resolution and a higher range of elements can be detected. Segmentation of regions in reconstructed micro/nanotomography images with low contrast also becomes possible if similar regions can be identified using SEM backscattered imaging and EDS. In this work we have developed a step-by-step correlative characterization protocol that involves 3D characterization of geological samples using micro and nano X-ray synchrotron tomography at the Advanced Light Source (ALS), Lawrence Berkeley National Laboratory, USA, coupled with SEM characterization for the identification of the elemental composition of the different mineral phases of the same features visible across all three characterization techniques. This protocol is broadly applicable for samples that require hard and tender X-rays for imaging.

## 2. Methods

Two rock samples were used in the multi-resolution characterization study: (i) a basaltic rock sample from the CarbFix project site in Iceland (Gislason *et al.*, 2010 ▸) and (ii) a serpentinite sample from the Bartlett Springs Fault complex in Northern California, USA. First, a piece of the basaltic rock and a 1 mm-diameter core of the serpentinite sample were fixed to an SEM sample stub using ep­oxy. The correlative study on both the sample was performed in the following steps: (i) microtomography of the samples, (ii) sample preparation for nanotomography using laser milling, (iii) nanotomography of both samples, (iv) samples removed from the SEM stub, mounted in ep­oxy and polished and (v) SEM characterization of the samples.

### 2.1. X-ray microtomography

The basalt sample mounted on the SEM sample stub is a piece of irregular shaped rock that looks roughly pyramidal in shape. The sample was mounted on a sample holder and placed in the hutch of the 8.3.2 microtomography beamline at ALS (MacDowell *et al.*, 2012 ▸). The beamline uses a multilayer (W/B_4_C, *d* = 2 nm with 1% bandpass. A monochromatic beam of 25 keV was used to scan the sample with 0.25 s of exposure per projection. 1500 projections were collected over 180° of stage rotation. 1500 projections were found to be adequate in obtaining data of high quality as the sample did not take up the entire horizontal FOV. Fifteen dark and flat-field images were collected at the end of the scan for background subtraction during reconstruction. The projections were collected using a PCO.edge sCMOS camera with a 50 µm LuAG:Ce scintillator, using a 10× Olympus objective lense. The source-to-sample distance was ∼20 m and the sample-to-detector distance was 17.08 mm. The pixel size of the projections collected was 0.65 µm.

The serpentinite sample was a ∼1 mm drill core mounted on the SEM stub in an upright position. Similar to the basalt sample the serpentinite sample was scanned at 25 keV, and 1500 projections were collected at 0.25 s of exposure per projection over 180°.

In order to maintain the same alignment for the samples during microtomography, laser milling and nanotomography 0 and 90° are marked on the SEM stub and visually aligned such that the 0° mark on the sample stub is parallel to the beam while placing the sample on the beamline stage.

Reconstruction of the two datasets was performed using the National Energy Research Scientific Computing (NERSC) supercomputing facility located at Lawrence Berkeley National Laboratory. A reconstruction code based on *TomoPy* (Gürsoy *et al.*, 2014 ▸) which uses the Gridrec algorithm (Dowd *et al.*, 1999 ▸), that has been adapted for the beam and data collection parameters used at ALS, was used and 2160 reconstructed slices per dataset in the form of tiff images were obtained. A non-commercial version of *Dragonfly ORS* software was used for image segmentation, visualization and volumetric measurements of the data (https://www.theobjects.com/dragonfly).

### 2.2. Sample preparation for X-ray nanotomography

Due to the specific setup of the tender X-ray nanotomography beamline at the ALS, the FOV is ∼70 µm × 70 µm (Nichols *et al.*, 2022 ▸). Thus, the sample size in order to fit within the FOV must either have a diameter of 50–60 µm or the longest horizontal dimension should be 50–60 µm. In certain hard X-ray beamlines at other synchrotron facilities around the world, it is sometimes possible to scan samples much larger than the FOV (Okuma & Wakai, 2024 ▸). This requires identifying and centering the region of interest (ROI) within the FOV and performing local tomography. Since the nanotomography beamline at the ALS operates in the tender range of 6–15 keV, scanning samples with higher density that are significantly larger than the FOV is not possible. In such cases there are three sample preparation methods that are commonly employed: (i) performing a focused ion beam (FIB) liftout in the shape of a cube in SEM, (ii) preparing a pillar of the sample using laser milling and (iii) mechanically polishing the sample to a tip that fits within the FOV. Each of these techniques have their own merits and disadvantages. FIB liftout can be performed at the precise ROI if it is visible on the surface of the sample (White *et al.*, 2019 ▸). The liftout has a polished surface on which complementary analysis techniques such as EDS to obtain the elemental composition and electron backscatter diffraction (EBSD) to obtain the crystallographic grain structure (Garum *et al.*, 2021 ▸) can be performed. Nano-CT sample liftout with FIB, although it is precise, requires significant time for sample preparation per sample and can also be restrictive in terms of access to a FIB-equipped SEM and expertise in the usage of FIB. On the other hand, nano-CT sample preparation using laser milling requires much less time but is also less precise in terms of the final sample containing the ROI (Bailey *et al.*, 2017 ▸). Thus, it is more suitable for samples with a certain degree of homogeneity. The third method of nano-CT sample preparation is by polishing the sample to a tip. This is the most accessible method of sample preparation as it requires the least expertise in nano-CT sample preparation and requires the least amount of time. However, it is the least precise method and the final sample will have varying diameter which can cause challenges during the final image segmentation or if a sub-volume visualization of the data is desired. In order to prepare a nanotomography sample with high degree of precision when the desire is to image a very specific ROI, some combination of the above three techniques can be employed. For example, for high rate of material removal laser milling or mechanical polishing can initially be employed followed by FIB liftout once proximity to the ROI has been achieved.

**Figure 1:**
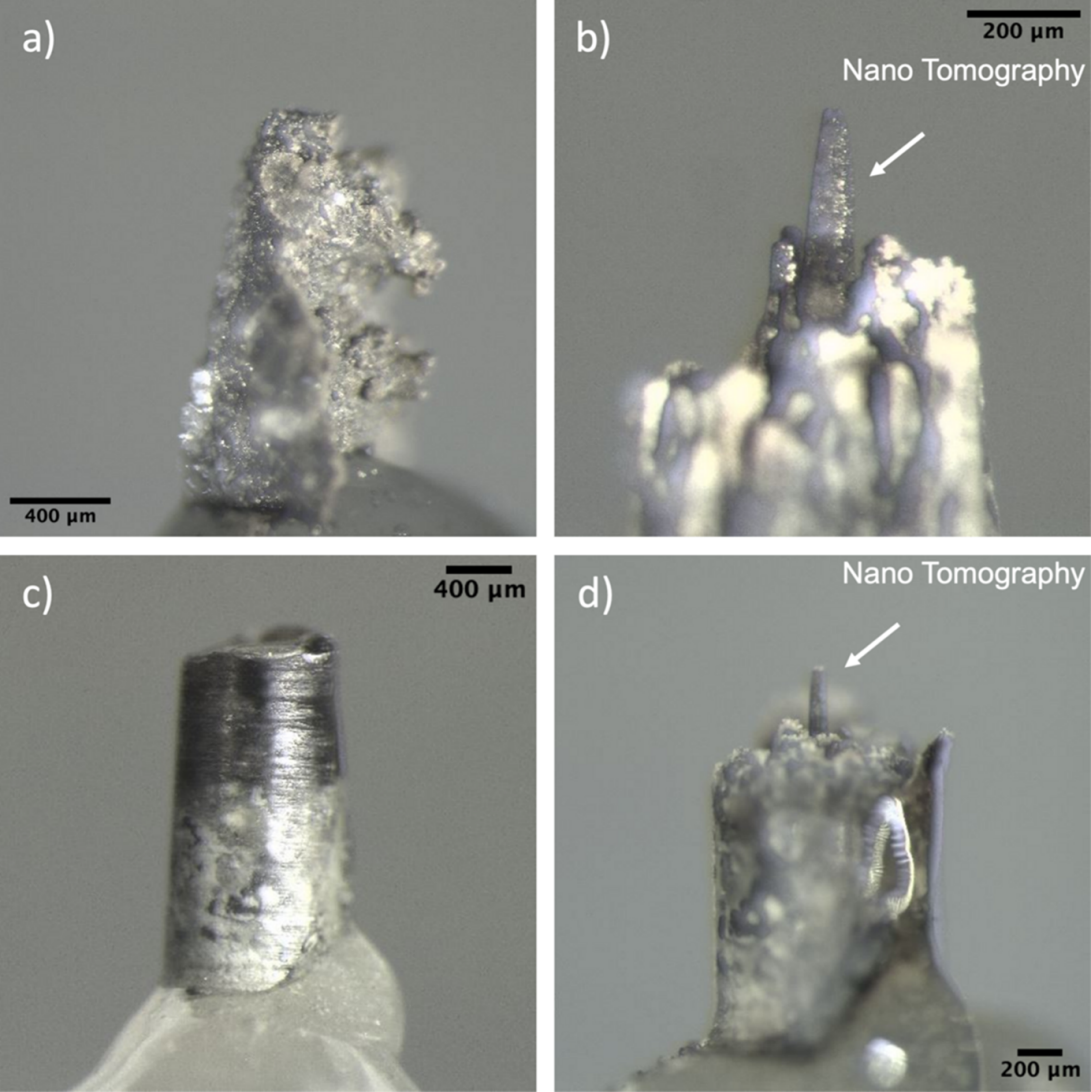
Optical images of (*a*) basalt microtomography sample, (*b*) basalt nanotomography sample, (*c*) serpentinite micro-CT sample and (*d*) serpentinite nano-CT sample.

For preparation of the nano-CT samples in the current study, we used laser milling as it provides the most uniform sample in the least amount of time among the various other techniques. Once a specific ROI was identified from the micro-CT data, the sample was placed inside the milling chamber of a Oxford Lasers Alpha 532 laser milling system. The 0 and 90° already marked on the SEM stub holding the sample helps to align the sample and position the laser for the desired ROI. The laser milling system uses a 532 nm laser with 50 µm laser diameter at focus. In order to create a 150–200 µm-long pillar out of the samples, an array of concentric circular milling patterns with 50 µm difference between subsequent patterns was used. For milling of geological samples, a laser with 98% laser power and 0.1 mm s^−1^ speed was found to gives the optimal results. In order to create a pillar having a length of 150–200 µm out of the ∼1 mm samples, ten passes of a circular pattern was found to mill to the required depth as shown in Figs. 1 ▸(*b*) and 1(*d*). As the focus of the laser beam changes with increasing depth, a small amount of tapering occurs in the sample pillar near the top. The milling was started with an initial circular pattern with 1 mm diameter and the diameter of the subsequent circular patterns were decreased by 50 µm with the final pattern having a diameter of 100 µm. This milling recipe produced nano-CT samples with 30–50 µm diameter which fits comfortably withing the FOV of the nano-CT microscope. The final surface of the nano-CT sample pillar does have some roughness which varies depending on laser ablation of the sample. The basalt nano-CT sample had a much smoother surface when compared with that of the serpentinite sample. Also, it is not essential to have an initial core of the sample in order to prepare a nano-CT sample by laser milling as the basalt micro-CT sample was irregularly shaped whereas the serpentinite sample was a drill core, as shown in Figs. 1 ▸(*a*) and 1(*c*).

### 2.3. X-ray nanotomography

Nanotomography of the basalt and serpentinite samples prepared via laser milling was performed using the tender nano X-ray tomography beamline 11.3.1 (Nichols *et al.*, 2022 ▸) at the ALS. Illumination has since been improved to four-pole illumination from the original two-pole described by Nichols *et al.* (2022 ▸). The beamline uses a channel-cut Si 〈111〉 system, with an energy resolution of ∼1000. The samples were placed on the rotating stage inside the hutch with the 0° mark parallel to the beam at the beamline for the scan, and the hutch was closed for an hour for the temperature inside the hutch to stabilize. The distance from source to sample is ∼14 m and from sample to detector is 1.5 m. By observing sample radiographs it was determined that the optimal X-ray absorption for imaging of the basalt sample was 7.5 keV and that of the serpentinite sample was 7 keV where both types of samples had ∼30% absorption of the beam in the radiographs.

For each sample 512 projections were collected with 5 s exposure time per projection over 180° sample rotation. The pixel size of the projections was 100 nm. The total scan time was ∼50 min. Fifteen dark and flat-field images for normalization of the projections during reconstruction were collected at the end of the scan with a PCO.edge sCMOS camera with a CsI(Tl) scintillator fitted on a 10× objective lens. Beamline 11.3.1, being a bend magnet beamline with Si 〈111〉 monochromator, requires longer exposure times to obtain quality data. As such, ∼50 min of scan time with 5 s exposure and 512 projections was found to be adequate for a high-quality reconstruction with the least amount of noise. Increasing the exposure time slightly reduces the noise but increasing the number of projections did not significantly improve the image quality of the reconstructed data. Increasing the number of projections also creates the possibility of sample motion due to thermal variations in the hutch over the course of a long scan time, which introduces motion artifacts in the reconstructed data. Reconstruction of the nano-CT data was performed using the NERSC supercomputing facility with a modified reconstruction algorithm based on *TomoPy*, which uses the Gridrec algorithm (Dowd *et al.*, 1999 ▸), that has been adapted for the beam and data collection parameters used at ALS. *Dragonfly ORS* was used for image segmentation and 3D visualization of the data.

### 2.4. Sample preparation for SEM analysis

**Figure 2:**
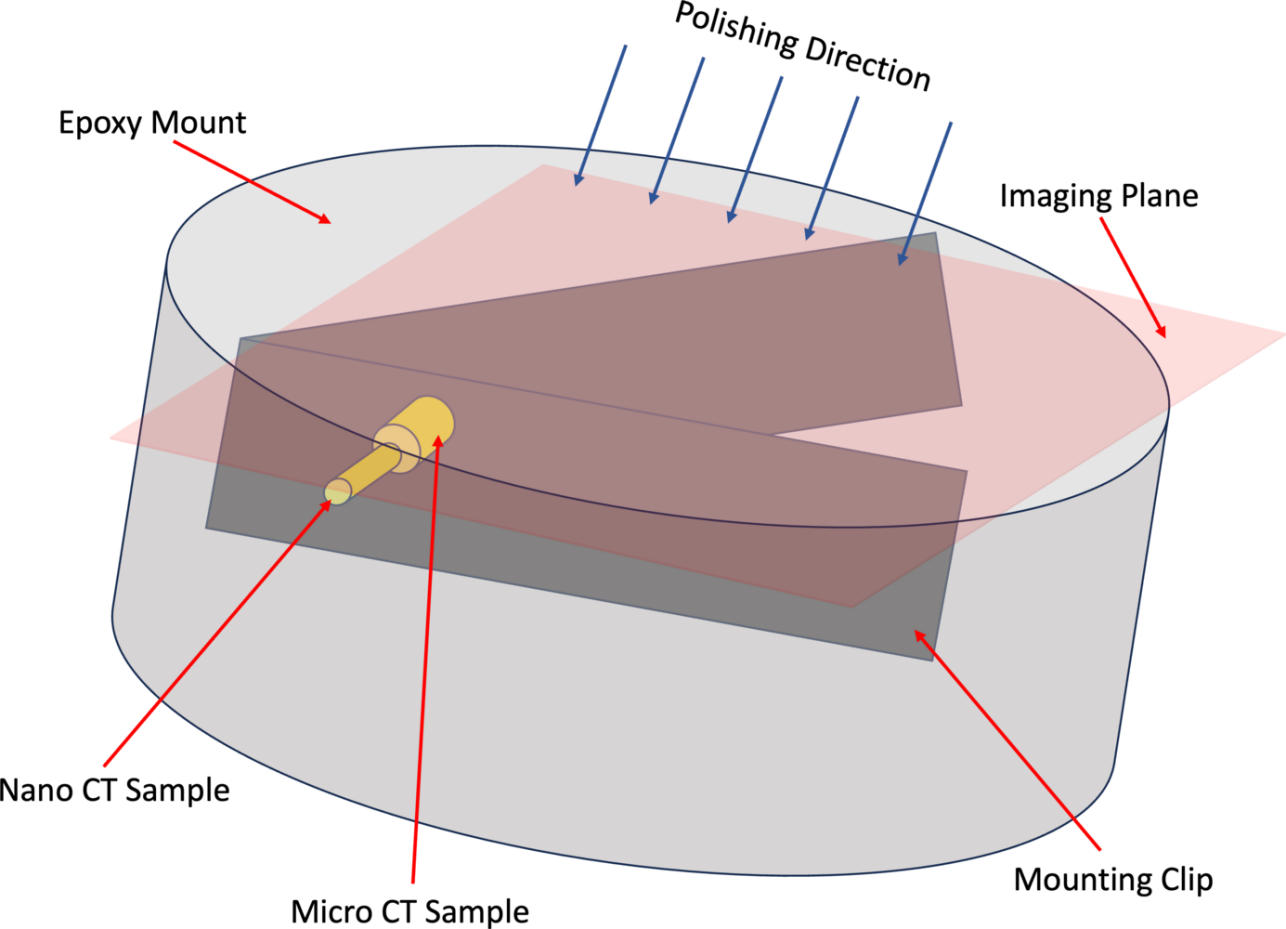
Schematic representation of sample mounting and sectioning for SEM analysis.

Once the 3D microstructure of the sample from both micro-CT and nano-CT was obtained, both samples were mounted in ep­oxy in a specific orientation. The sample, which was attached to the SEM stub, was gently removed and attached to a piece of stainless steel foil with the 0° mark parallel to the polishing direction using ep­oxy. The stainless foil acts as a mounting clip to hold the sample in the horizontal position as shown in Fig. 2 ▸. The mounting clip along with the sample is then mounted in ep­oxy for polishing and sectioning. The ep­oxy required 8 h of curing time after which the sample was polished such that by the final polishing step the nano-CT sample pillar on top of the micro-CT sample gets sectioned horizontally in half and the microstructure from both the regions is visible for SEM analysis (indicated by the imaging plane in Fig. 2 ▸); 240, 320, 400, 600 and 800 grit polishing paper were used to expose parts of the micro- and nano-CT regions of both the samples on the surface followed by 6, 3 and 1 µm polishing fluid, and then by 0.05 µm alumina as the final polishing step. The samples were hand polished to have greater control over the process.

### 2.5. SEM imaging and EDS elemental mapping

Once the samples were polished and the desired imaging plane was achieved, they were coated with conductive gold for SEM imaging. SEM analysis was performed using a Zeiss EVO MA-10 SEM that is equipped with a backscatter electron (BSE) detector and an Oxford EDS detector for phase-contrast imaging and elemental mapping, respectively. An electron beam with 20 keV accelerating voltage and 2 nA current was used for BSE imaging of the whole sample before focusing on a specific region containing only microtomography and another only nanotomography samples. EDS elemental mapping was performed on both the regions and point scans were performed on regions with differing contrast to identify the stoichiometry of the different phases and for their subsequent identification.

## 3. Results

X-ray microtomography was performed on a piece of basaltic rock and a 1 mm drill core of serpentinite rock sample to obtain the 3D microstructure with 650 nm pixel size. An ROI was identified in the samples from the micro-CT data, and to resolve the 3D microstructure of finer features in the ROI of both samples nano X-ray tomography was performed. The pixel size for nanotomography was 100 nm. The elemental composition of the phases observed in both micro- and nano-CT was obtained using EDS.

### 3.1. Basalt

#### 3.1.1. X-ray microtomography

**Figure 3:**
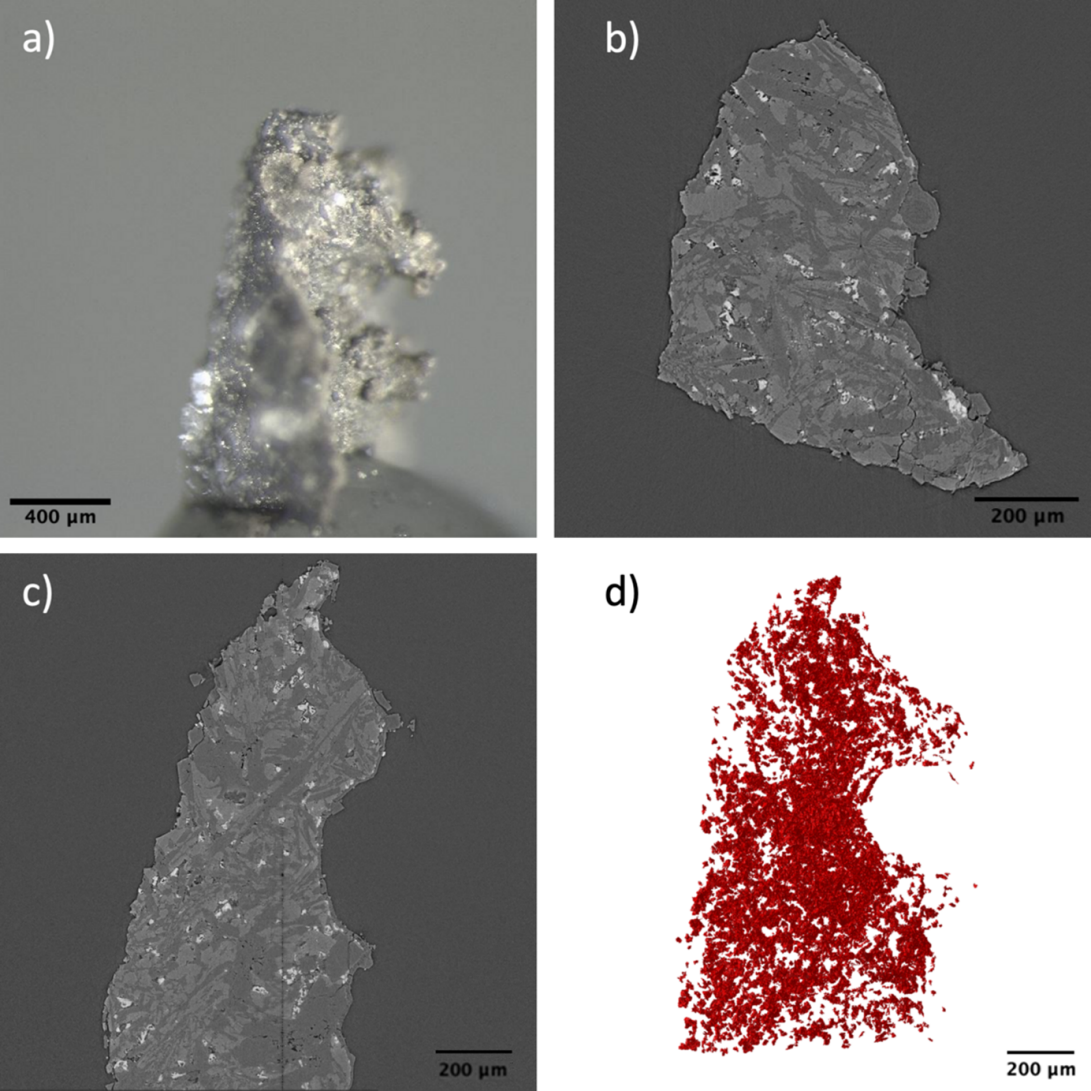
(*a*) Optical image of the basalt micro-CT sample. (*b*) Vertical and (*c*) horizontal slice of the basalt micro-CT reconstructed data. (*d*) 3D visualization of the bright particles.

The piece of basaltic rock used in this study had an irregular shape but appeared roughly pyramidal in an upright position as shown in Fig. 3 ▸(*a*). The specimen was obtained from the CarbFix site, a pilot carbon mineralization facility in Iceland (Gislason *et al.*, 2010 ▸). The pore structure of the sample becomes highly relevant for understanding reactive flow and ultimate carbon budget within the system. Along with pores, it is also important to understand the specific phases in which pores occur as certain phases are more favorable for CO_2_ mineralization to occur. Looking at the vertical and horizontal slices of the micro-CT reconstructed data, as shown in Figs. 3 ▸(*b*) and 3(*c*), respectively, the specimen was observed to contains at least three different phases and small amounts of detected porosity. The three phases exhibit different gray levels based on their relative densities. The densest phase appears as the brightest regions followed by the phase with medium density as light gray, and finally the lowest density phase appears as dark gray regions in the reconstructed images. The sample consisted of mostly the dark and light gray phase with the light gray region forming the bulk or matrix of the sample. The light gray phase was found to have a higher volume fraction of ∼63% compared with the 41% of the dark gray regions. The dark and light gray regions were later identified to be feldspar and pyroxene, respectively, from EDS analysis. Within the light and dark regions, bright particles can also be seen. These small particles are homogeneously distributed in the whole specimen as shown in the 3D visualization in Fig. 3 ▸(*d*). Given the size of these particles it is difficult to obtain details of the 3D structure of these particles from the micro-CT data. Thus, a region close to the tip of the sample was found to be a suitable location for 3D imaging using nano-CT.

#### 3.1.2. X-ray nanotomography

**Figure 4:**
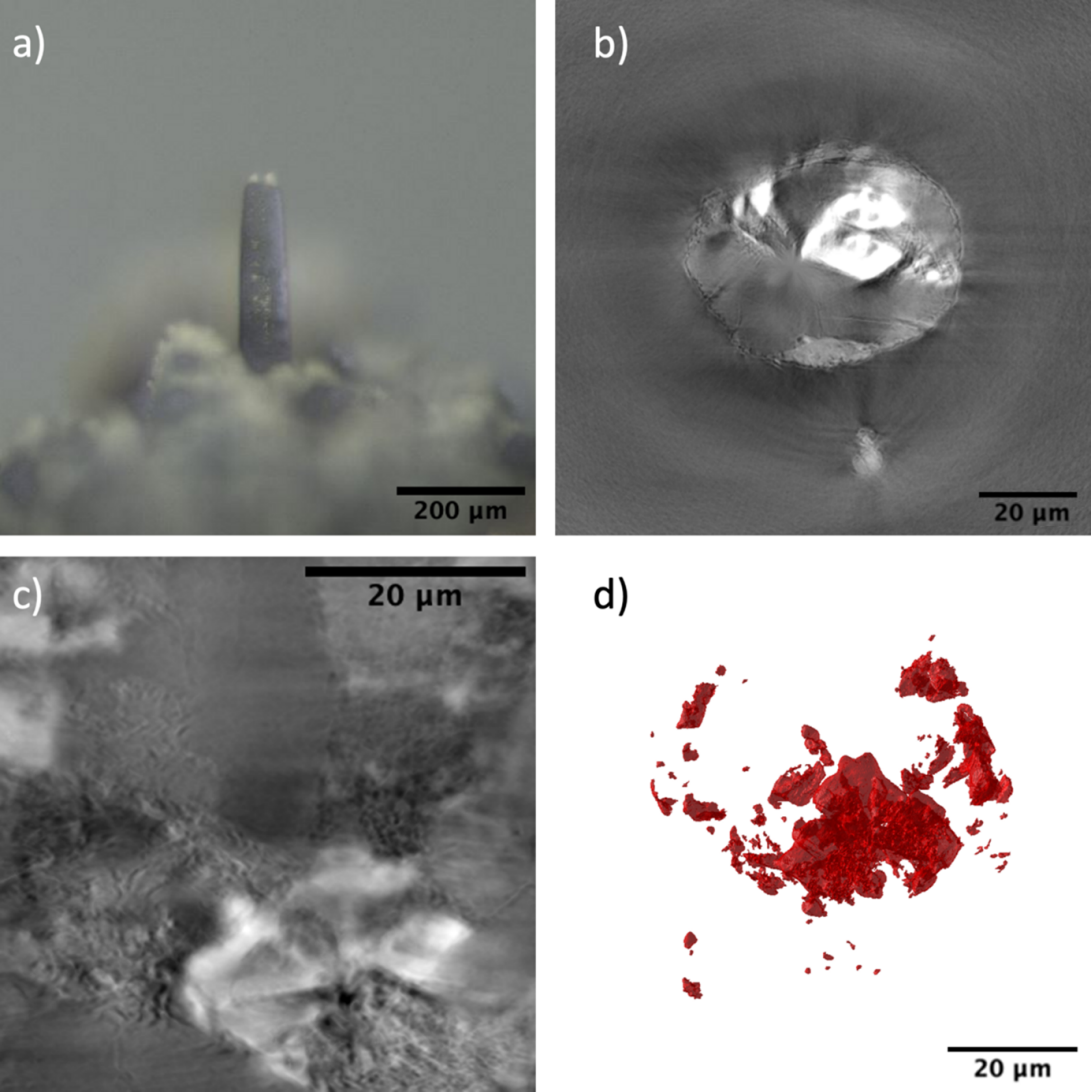
(*a*) Optical image of the basalt nano-CT pillar sample. (*b*) Vertical and (*c*) horizontal slice of the basalt nano-CT reconstructed data. (*d*) 3D visualization of the bright particles.

After preparing the nano-CT sample near the top of the micro-CT sample using laser milling, nano-CT data were obtained for a 70 µm-long cylindrical pillar having a diameter of ∼40 µm as shown in Fig. 4 ▸(*a*). The sample appears to have a far more complex structure when looked at with high-resolution nanotomography than what can be resolved by micro-CT. There appears to be some light and dark gray regions even inside the bright particle along with some porosity as shown in Figs. 4 ▸(*b*) and 4(*c*). The bright particle itself is mostly spherical in shape, containing light and dark gray mineral phases at its center. There were a few other bright particles that were present in the nano-CT sample, which were mostly elongated in shape and smaller in size when compared with the largest particle, perhaps indicative of incipient skeletal crystals, as shown in Fig. 4 ▸(*d*). Since the X-ray nano-CT data were collected at 7.5 keV, which is above the X-ray absorption edge of Fe at 7.1 keV, the brightness of these particle regions (indicative of higher absorption) is possibly due to high Fe concentrations in the particles.

#### 3.1.3. SEM

**Figure 5:**
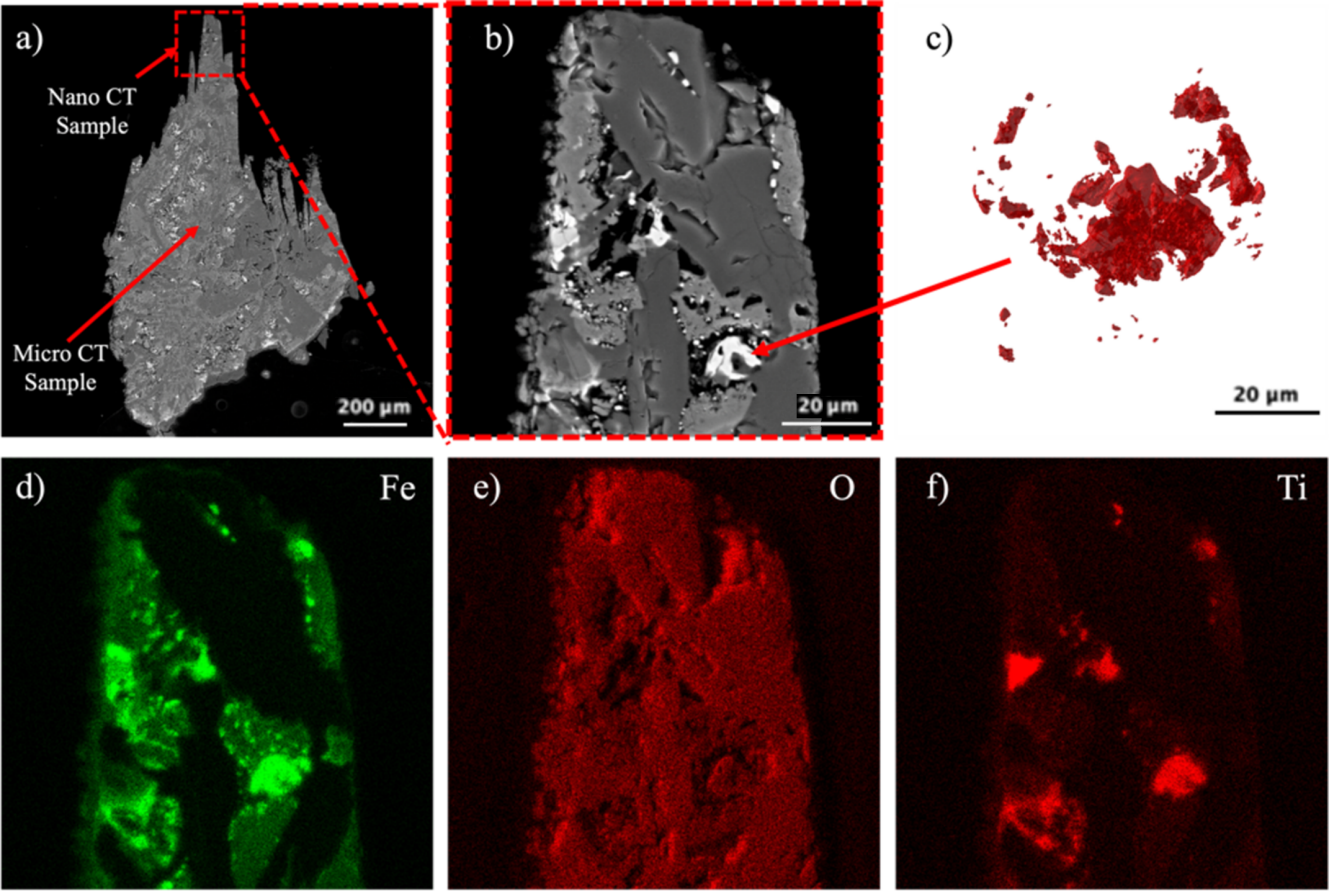
SEM BSE image of (*a*) the micro- and nano-CT sample and (*b*) the nano-CT sample region. (*c*) 3D visualization of the ilmenite particles from nano-CT data. Also, EDS composition maps of the nano-CT sample region for (*d*) Fe, (*e*) O and (*f*) Ti.

To understand the chemical composition of the mineral phases in the micro- and nanotomography regions of the sample, it was mounted and polished until both the regions of micro- and nanotomography were in the imaging plane. In the case of the basalt sample, polishing was performed until a large particle of ilmenite was visible and on the imaging plane. Low-magnification imaging was performed to image the micro- and nanotomography regions together as shown in Fig. 5 ▸(*a*), after which the micro- and nano-CT regions were separately imaged with high magnification to correlate the features with micro- and nanotomography data, respectively, as shown in Figs. 4 ▸(*b*) and 4(*c*). EDS analysis provided the stoichiometry of the three different mineral phases present in the sample, namely feldspar, pyroxene and ilmenite. Based on the chemical composition and stoichiometry, the particle, as shown in Fig. 4 ▸(*d*), was determined to have a chemical composition of FeTiO_3_. Other bright regions of ilmenite also had similar stoichiometric ratios. The high-resolution SEM image of the nano-CT region in Fig. 4 ▸(*b*) also shows the inside of the large ilmenite particle containing a dark gray region of feldspar, pyroxene and some porosity. This aided in the segmentation of nano-CT data where the difference of intensity of the dark gray pyroxene and light gray feldspar and porosity at the center of the particle is not profound as shown in Figs. 4 ▸(*b*) and 4(*c*).

### 3.2. Serpentinite

#### 3.2.1. X-ray microtomography

**Figure 6:**
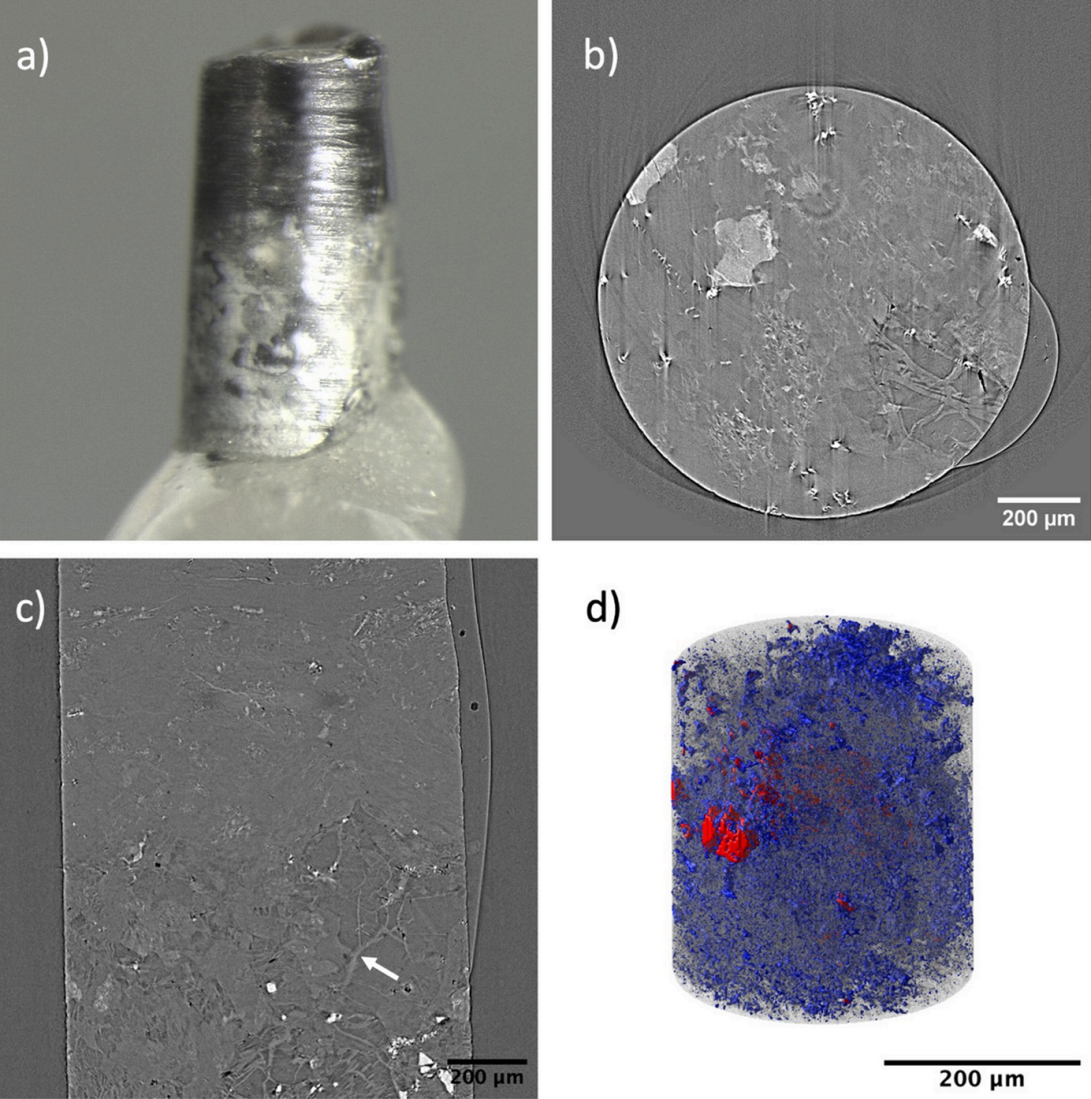
(*a*) Optical image of the serpentinite 1 mm core sample for micro-CT. (*b*) Horizontal and (*c*) vertical slice of the reconstructed micro-CT data. Also, (*d*) 3D visualization of the three mineral phases in the sample with gray, blue and red representing dark gray, light gray and bright mineral phases, respectively. The white arrow in (*c*) marks the serpentine veins.

3D microstructural characterization of the serpentinite sample was performed using micro-CT on a 1 mm core as shown in Fig. 6 ▸(*a*). From the horizontal slice of the reconstructed data, three different phases were identified based on the imaging contrast as shown in Fig. 6 ▸(*b*). Similar to the basalt sample the densest phase in the serpentinite sample appears as the brightest regions followed by the phase with medium density as light gray and finally the lowest density phase appears as dark gray regions in the reconstructed images. The majority of the sample consists of a dark gray mineral phase which forms the matrix of the sample. The sample also contains a sporadically distributed light gray phase in regions of 100–200 µm in size. The third phase of the sample is particles of a bright phase. The density of these particles is higher towards the bottom of the sample compared with the top region. The exact morphology of the bright particles is difficult to analyze from the micro-CT data. The sample also forms some root-like structure towards the bottom of the sample as shown in Fig. 6 ▸(*c*), marked with a white arrow. These structures are from entirely within the dark gray region and are possibly serpentinite veins (Andreani *et al.*, 2007 ▸). The sample is ∼74% dark gray phase, 24% light gray phase and 2% bright particle phase in terms of volume fraction. 3D visualization of the three mineral phases was done on a cylindrical sub-volume of the sample with 350 µm length and 300 µm diameter, as shown in Fig. 6 ▸(*d*) where the light gray phase is shown in blue, bright particles are shown in red, and the matrix (dark gray phase) of the sample is shown in gray.

#### 3.2.2. X-ray nanotomography

**Figure 7:**
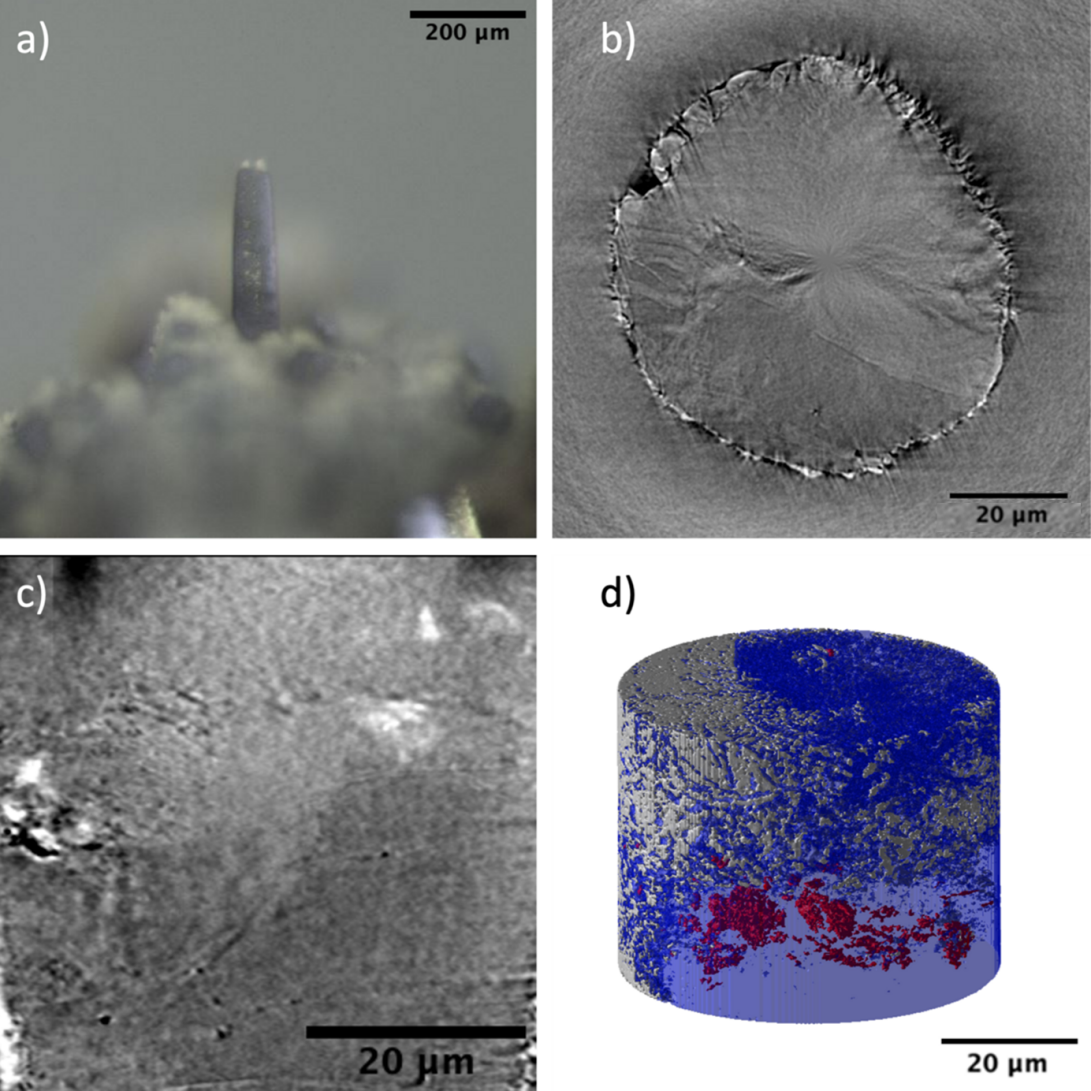
(*a*) Optical image of the serpentinite pillar sample for nano-CT. (*b*) Horizontal and (*c*) vertical slice of the reconstructed nano-CT data. Also, (*d*) 3D visualization of the three mineral phases in the sample with gray, blue and red representing dark gray, light gray and bright mineral phases, respectively.

From the micro-CT data, the ROI was identified to be the interface between the top half of the sample and the bottom half of the sample which contained the root-like structures. As the ROI is ∼600 µm away from the sample surface, three successive nano-CT samples were prepared as the depth of laser milling is ∼200 µm for each sample. Each of the two initial samples after preparation was scanned and the sample surface was made flat by grinding, and the third and final sample was used for SEM imaging as shown in Fig. 7 ▸(*a*). The volume fractions of the intermediate density phases were reversed in the nano-CT data. The nano-CT ROI contains ∼70% of the light gray phase and ∼28% of the dark gray phase. This indicates that the sample was prepared in a region containing one of the ∼200 µm regions of light gray phase as shown in the micro-CT reconstructed slice in Fig. 6 ▸(*b*). Some of the root-like structures were visible in the nano-CT data although the majority were outside the FOV. The visualization of the light and dark gray regions and the distribution of the bright particles towards the bottom of the sample is shown in Fig. 7 ▸(*d*) where gray, blue and red represents dark gray, light gray and bright particle mineral phases, respectively.

#### 3.2.3. SEM

**Figure 8:**
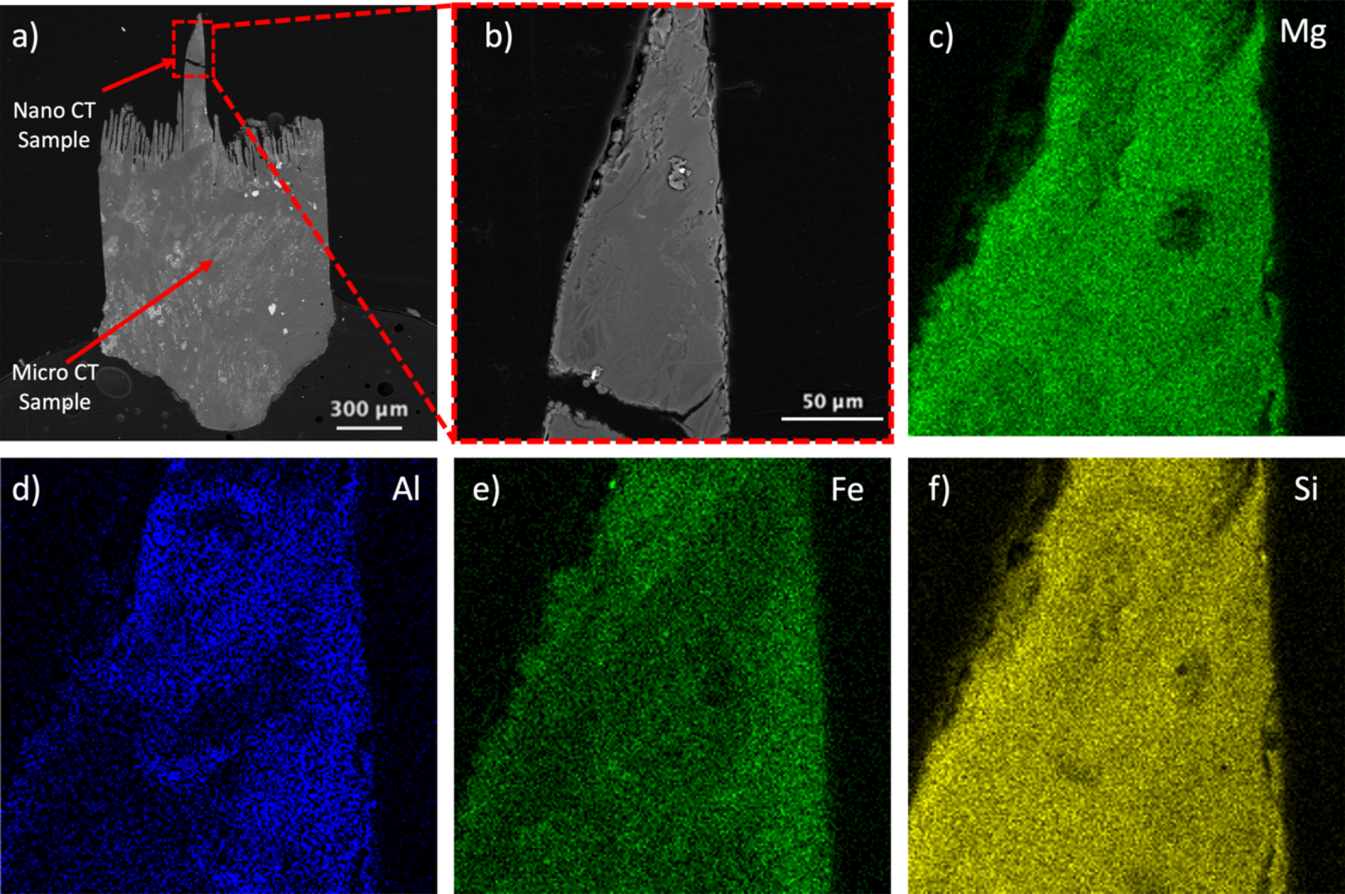
SEM BSE image of (*a*) the micro- and nano-CT sample and (*b*) the nano-CT sample region. Also, EDS composition maps of the nano-CT sample region for (*c*) Mg, (*d*) Al, (*e*) Fe and (*f*) Si.

After scanning the third nano-CT sample it was mounted in ep­oxy and polished until both the nano-CT and micro-CT samples were on the same imaging plane, as shown in Fig. 8 ▸(*a*). The light gray and dark gray regions along with the bright particles are visible in the BSE image as shown in Fig. 8 ▸(*b*). From the EDS data the light and dark gray regions were ascertained to be chlorite and serpentinite mineral phases, respectively, and the bright particle phase was found to be magnetite. Comparing this image with the EDS Fe and Al concentration map, as shown in Fig. 8 ▸(*e*), it was found that the light gray region of chlorite contains high concentrations of Fe and Al. Si and Mg concentrations are not directly related to the contrast between the two mineral phases, as shown in Figs. 8 ▸(*c*), 8(*d*) and 8(*f*), but Al and Mg were found to have lower concentrations in regions that contained the root-like structures. Point scans performed on these structures and the matrix also indicate this difference. Further analysis and characterization are needed to fully resolve the chemical differences between these structures and process of formation using nano-CT and XRD.

### 3.3. Discussion

Characterization of the two samples using the correlative microscopy protocol provided insight into the mineral and porosity distribution in the overall sample and how the distribution changes at a fine scale in a particular ROI. Although elemental distribution by EDS is not a 3D technique, since SEM imaging of a specific cross section of the sample is achievable by employing the sectioning process mentioned in Section 2.4 it is possible to correlate the minerals identified by EDS and backscattered contrast with the contrast in the reconstructed tomography slice. For example, the light and dark gray regions as shown in the reconstructed nano-CT slice in Fig. 7 ▸(*c*) can also be seen in the backscattered image in Fig. 8 ▸(*b*). The cause of the contrast between the light and dark regions is a result of the dark gray region being serpentinite and the light gray region being chlorite as revealed in the EDS Fe- and Al-distribution map of the sample where the light gray chlorite region in the sample contains much higher concentrations of Fe and Al. Phyllo­silicates such as chlorite and serpentinite are known to co-exist (Andreani *et al.*, 2007 ▸) where serpentinite transforms into chlorite by changing the structure of metal-oxide tetrahedral and octahedral layers (Banfield & Bailey, 1996 ▸). When occurring together in clay, dissolution of serpentinite forms smectite whereas chlorite transforms into vermiculite which then transforms into smectite (Zhang *et al.*, 2021 ▸). In the case of chlorites, the Fe:Mg ratio is highly important in identifying the specific member of the chlorite family, where the end-members are Fe-rich chamosite and Mg-rich clinochlore (Lee *et al.*, 2003 ▸). EDS point scans performed on the chlorite region of the sample show 20.16 ± 0.13 at% Mg and 4.22 ± 0.09 at% Fe which indicates the chlorite to be possibly clinochlore. Based on this the 3D distribution of the two mineral types was acquired from the micro-CT data and the fine-scale 3D structure of the minerals was revealed by the nano-CT of the sample. The sample is mostly serpentinite with discrete regions of chlorite and magnetite distributed throughout the sample.

Using this correlative microscopy protocol for samples containing fine-scale distributed structures, information regarding the sample is extracted in three stages: (i) X-ray micro-CT provides the statistical analysis of the distribution of the fine structures, (ii) X-ray nano-CT reveals the high-resolution morphology of this structures and (iii) SEM-EDS provides the elemental composition. Combining these three microscopy techniques at the same ROI creates a powerful multimodal and multiscale microscopy technique for the analysis of geological specimens. For example, ilmenite particles were found to be distributed throughout the basalt sample as shown in Fig. 3 ▸(*d*). Although the rough shape of these particles can be understood from the micro-CT data, higher-resolution imaging is required to fully resolve their structure. Nano-CT imaging on the same sample shows one such particle to be roughly spherical in shape as shown in Fig. 5 ▸(*c*). However, the particle also contains regions of feldspar and pyroxene at its core. At this scale, it becomes clear that the ilmenite habit is skeletal with intergrowths of plagioclase (feldspar) and augite (pyroxene). Such crystal habitats are known to form due to rapid cooling and crystallization. The existence of Ti-rich pyroxene and ilmenite indicates the formation of the basalt from a Ti-rich parent magma (Worden *et al.*, 2020 ▸). Olivine and ilmenite form first during the initial stages of cooling followed by pyroxene and feldspar inside the spherical particle. Later olivine is converted to pyroxene forming lath-like structures in the overall sample (Snyder *et al.*, 1992 ▸; Roedder & Weiblen, 1970 ▸; James & Jackson, 1970 ▸). The elemental composition of the mineral particle and the phases contained within it were obtained by analyzing a cross section of the same mineral particle using EDS. Also, the 3D data from nano-CT as well as the backscattered image of the sample cross section reveals the presence of finer ilmenite particles which are not detectable in micro-CT data. Thus, adopting this correlative protocol at the ALS and other light sources around the world would enable multiscale and multimodal characterization of geological and material science samples.

## 4. Conclusion

A correlative protocol was developed and adopted for performing micro and nano X-ray tomography with SEM analysis for the microstructural and compositional analysis of geological samples at the Advanced Light Source. Two rock samples of basalt and serpentinite were analyzed using this protocol. Hard X-ray micro-CT was initially performed on the two samples, one of which was a ∼1 mm core of serpentinite and the other was an irregularly shaped piece of basalt, to analyze the 3D microstructure of these rocks. Based on the imaging contrast from the micro-CT reconstructed images three different phases were found to exist in the basalt sample. One of these phases was ilmenite in the form of particles homogeneously distributed in the sample. Similarly, for the serpentinite sample three phases were also found to exist in the sample, with serpentinite vein formations within the serpentine phase. To analyze the morphology of the ilmenite particles in the basalt sample and the interface of the root-like structures in the serpentinite sample, tender X-ray nano­tomography was performed on sample pillars of both types of samples, prepared via laser milling. SEM imaging and EDS analysis were performed on both micro- and nanotomography regions to determine the chemical composition of not just the entire nano- and micro-CT regions but also specifically of features visible in micro- and nano-CT-reconstructed data. Using this protocol, we were able to track features and ROIs across the three different techniques.

## References

[bb1] Andreani, M., Mével, C., Boullier, A. & Escartín, J. (2007). *Geochem. Geophys. Geosyst.* **8**, 2006GC001373.

[bb2] Bailey, J. J., Heenan, T. M. M., Finegan, D. P., Lu, X., Daemi, S. R., Iacoviello, F., Backeberg, N. R., Taiwo, O. O., Brett, D. J. L., Atkinson, A. & Shearing, P. R. (2017). *J. Microsc.* **267**, 384–396. 10.1111/jmi.12577 PMC6849567 28504417

[bb3] Banfield, J. F. & Bailey, S. W. (1996). *Am. Mineral.* **81**, 79–91.

[bb4] Bellet, D., Gorges, B., Dallery, A., Bernard, P., Pereiro, E. & Baruchel, J. (2003). *J. Appl. Cryst.* **36**, 366–367.

[bb6] Borsato, A., Frisia, S., Howard, D. & Greig, A. (2021). *At. Spectrosc.* **185**, 106308.

[bb7] Buffiere, J., Maire, E., Adrien, J., Masse, J. & Boller, E. (2010). *Exp. Mech.* **50**, 289–305.

[bb8] Cai, B., Wang, J., Kao, A., Pericleous, K., Phillion, A. B., Atwood, R. C. & Lee, P. D. (2016). *Acta Mater.* **117**, 160–169.

[bb9] Chen, J. R., Chao, E. C. T., Back, J. M., Minkin, J. A., Rivers, M. L., Sutton, S. R., Cygan, G. L., Grossman, J. N. & Reed, M. J. (1993). *Nucl. Instrum. Methods Phys. Res. B*, **75**, 576–581.

[bb10] Cnudde, V. & Boone, M. N. (2013). *Earth-Sci. Rev.* **123**, 1–17.

[bb5] Dowd, B. A., Campbell, G. H., Marr, R. B., Nagarkar, V. V., Tipnis, S. V., Axe, L. & Siddons, D. P. (1999). *Proc. SPIE*, **3772**, https://doi.org/10.1117/12.363725.

[bb13] Garum, M., Glover, P. W. J., Lorinczi, P., Scott, G. & Hassanpour, A. (2021). *Energy Fuels*, **35**, 702–717.

[bb14] Ghahari, S. M., Davenport, A. J., Rayment, T., Suter, T., Tinnes, J. P., Padovani, C., Hammons, J. A., Stampanoni, M., Marone, F. & Mokso, R. (2011). *Corros. Sci.* **53**, 2684–2687.

[bb15] Gislason, S. R., Wolff-Boenisch, D., Stefansson, A., Oelkers, E. H., Gunnlaugsson, E., Sigurdardottir, H., Sigfusson, B., Broecker, W. S., Matter, J. M. & Stute, M. (2010). *Int. J. Greenhouse Gas Contr.* **4**, 537–545.

[bb16] Goral, J., Andrew, M., Olson, T. & Deo, M. (2020). *Mar. Petrol. Geol.* **111**, 886–904.

[bb17] Gürsoy, D., De Carlo, F., Xiao, X. & Jacobsen, C. (2014). *J. Synchrotron Rad.* **21**, 1188–1193. 10.1107/S1600577514013939 PMC4181643 25178011

[bb18] Huang, T., Niverty, S., Sundar, A. & Chawla, N. (2023). *Mater. Charact.* **205**, 113331.

[bb20] James, O. B. & Jackson, E. D. (1970). *J. Geophys. Res.* **75**, 5793–5824.

[bb21] Lee, B. D., Sears, S. K., Graham, R. C., Amrhein, C. & Vali, H. (2003). *Soil Sci. Soc. Am. J.* **67**, 1309–1317.

[bb22] Lin, Z., Zhang, X., Nandi, P., Lin, Y., Wang, L., Chu, Y. S., Paape, T., Yang, Y., Xiao, X. & Liu, Q. (2024). *Commun. Biol.* **7**, 280. 10.1038/s42003-024-05950-y PMC10917812 38448784

[bb23] MacDowell, A. A., Parkinson, D. Y., Haboub, A., Schaible, E., Nasiatka, J. R., Yee, C. A., Jameson, J. R., Ajo-Franklin, J. B., Brodersen, C. R. & McElrone, A. J. (2012). *Proc. SPIE*, **8506**, 850618.

[bb24] Mathews, J. P., Campbell, Q. P., Xu, H. & Halleck, P. (2017). *Fuel*, **209**, 10–24.

[bb25] Mayo, S. C., Stevenson, A. W. & Wilkins, S. W. (2012). *Materials*, **5**, 937–965. 10.3390/ma5050937 PMC5458972 28817018

[bb26] Nichols, J. B., Voltolini, M., Gilbert, B., MacDowell, A. A. & Czabaj, M. W. (2022). *Rev. Sci. Instrum.* **93**, 023704. 10.1063/5.0076322 35232135

[bb27] Nikitin, V., Tekawade, A., Duchkov, A., Shevchenko, P. & De Carlo, F. (2022). *J. Synchrotron Rad.* **29**, 816–828. 10.1107/S1600577522003095 PMC9070713 35511014

[bb28] Okuma, G., Endo, M., Minagawa, H., Inoue, R., Kakisawa, H., Kohata, T., Osada, T., Yamamoto, T., Azuma, M., Takeuchi, A., Uesugi, M., Guillon, O. & Wakai, F. (2023). *Adv. Eng. Mater.* **25**, 2201534.

[bb29] Okuma, G. & Wakai, F. (2024). *J. Am. Ceram. Soc.* **107**, 1706–1724.

[bb30] Robinson, J. B., Brown, L. D., Jervis, R., Taiwo, O. O., Millichamp, J., Mason, T. J., Neville, T. P., Eastwood, D. S., Reinhard, C., Lee, P. D., Brett, D. J. L. & Shearing, P. R. (2014). *J. Synchrotron Rad.* **21**, 1134–1139. 10.1107/S1600577514014209 PMC4161039 25178003

[bb12] Roedder, E. & Weiblen, P. W. (1970). *Science*, **167**, 641–644. 10.1126/science.167.3918.641 17781528

[bb31] Snyder, G. A., Taylor, L. A. & Neal, C. R. (1992). *Geochim. Cosmochim. Acta*, **56**, 3809–3823.

[bb32] Stock, S. R. (2008). *Int. Mater. Rev.* **53**, 129–181.

[bb19] Taina, I. A., Heck, R. J. & Elliot, T. R. (2008). *Can. J. Soil. Sci.* **88**, 1–19.

[bb33] Takeuchi, A., Uesugi, K., Uesugi, M., Toda, H., Hirayama, K., Shimizu, K., Matsuo, K. & Nakamura, T. (2021). *Rev. Sci. Instrum.* **92**, 023701. 10.1063/5.0020293 33648114

[bb34] Wakabayashi, D., Suzuki, Y., Shibazaki, Y., Sugiyama, H., Hirano, K., Nishimura, R., Hyodo, K., Igarashi, N. & Funamori, N. (2022). *Rev. Sci. Instrum.* **93**, 033701. 10.1063/5.0070720 35365003

[bb35] Wang, Y. & Miller, J. D. (2020). *Earth-Sci. Rev.* **211**, 103406.

[bb36] White, R. T., Ramani, D., Eberhardt, S., Najm, M., Orfino, F. P., Dutta, M. & Kjeang, E. (2019). *J. Electrochem. Soc.* **166**, F914–F925.

[bb37] Worden, R. H., Utley, J. E. P., Butcher, A. R., Griffiths, J., Wooldridge, L. J. & Lawan, A. Y. (2020). *Geol. Soc. London Spec. Publ.* **484**, 189–204.

[bb38] Wu, T., Li, X., Zhao, J. & Zhang, D. (2017). *Water Resour. Res.* **53**, 5438–5450.

[bb39] Zhang, H., Gilbert, B. & Banfield, J. F. (2021). *Chem. Mater.* **33**, 6338–6345.

